# Association between oxidative balance scores and peripheral artery disease in US adults: a cross-sectional study

**DOI:** 10.3389/fnut.2024.1497784

**Published:** 2025-01-17

**Authors:** Min Zhou, Peng-Fei He, Keren Zhang, Li-Juan Deng, Ning Wang, Gang Wang, Guang-Yao Yang, Shang Ju

**Affiliations:** ^1^Department of Peripheral Vascular Surgery, Dongzhimen Hospital, Beijing, China; ^2^Beijing University of Chinese Medicine, Beijing, China; ^3^Dongfang Hospital, Beijing, China; ^4^Beijing Hepingli Hospital, Beijing, China

**Keywords:** peripheral artery disease, oxidative balance score, antioxidants and prooxidants, diet, lifestyle

## Abstract

**Background:**

The Oxidative Balance Score (OBS) quantifies the overall oxidative stress burden, with higher scores indicating greater antioxidant (relative to prooxidant) activity. This study aimed to examine the association between peripheral arterial disease (PAD) and OBS.

**Methods and materials:**

Data from the National Health and Nutrition Examination Survey (NHANES, 1999–2004) were analyzed for participants with ankle-brachial index (ABI) measurements. The total Oxidative Balance Score (OBS) comprised a lifestyle OBS (four lifestyle categories) and a dietary OBS (16 dietary factors). Logistic regression analyses evaluated associations between PAD and total OBS, lifestyle OBS, and dietary OBS. Restricted cubic spline (RCS) analyses assessed dose–response relationships between ABI, PAD, and OBS. Mediation analyses investigated the roles of glucolipid metabolism and renal function in the OBS-PAD association. Sensitivity and stratification analyses were conducted to ensure robustness.

**Results:**

This study included 2,437 eligible adult participants. Logistic regression analysis, adjusted for multiple potential confounders, revealed negative associations between lifestyle OBS (OR = 0.88; 95% CI: 0.79, 1.00), total OBS (OR = 0.97; 95% CI: 0.94, 0.99), and the likelihood of PAD (all *p* < 0.05). Restricted cubic spline (RCS) analysis demonstrated a linear relationship between total OBS and PAD, with the likelihood of PAD decreasing as total OBS increased *p* for nonlinearity = 0.736. Dietary OBS, lifestyle OBS, and total OBS all showed positive linear correlations with ABI levels (all *p* < 0.05). Mediation analysis indicated that fasting plasma glucose (FPG) and creatinine (CREA) mediated 5.9 and 0.8% of the association between total OBS and PAD, respectively (all *p* < 0.05). Sensitivity analyses confirmed the negative association between total OBS and PAD *p* < 0.05, supporting the stability of the results. Stratified analyses highlighted the significant influence of Age, particularly in the younger population aged 20–44 years, a group warranting greater attention.

**Conclusion:**

Our study demonstrated that higher total OBS is associated with a lower likelihood of PAD. Adopting an antioxidant-rich diet alongside a healthy lifestyle may help mitigate PAD risk. Additionally, modulating FPG and CREA levels could offer potential value in addressing the link between low OBS and PAD.

## 1 Introduction

Peripheral arterial disease (PAD) is an atherosclerotic condition affecting arteries outside the heart and brain, most commonly involving the lower limb arteries. It is characterized by high morbidity, disability, and mortality (1, 2). Major risk factors include age, diabetes, hyperlipidemia, hypertension, chronic renal insufficiency, and smoking, which contribute to PAD development by accelerating atherosclerosis and causing endothelial damage (3). Patients with PAD face a high risk of cardiovascular events, atrial fibrillation, deep vein thrombosis, and stroke, potentially due to endothelial dysfunction and systemic inflammation (4). Diagnosis relies on an ankle-brachial index (ABI) ≤0.9, which reflects the degree of arterial flow restriction in the lower extremities (5). The main objectives of PAD treatment are to slow down the progression of the disease, reduce the risk of cardiovascular events, alleviate painful symptoms, encourage the formation of collateral circulation, and decrease amputation rates. Current treatment options include lifestyle changes (such as quitting smoking and exercising), medications (such as antithrombotic, lipid-lowering, glucose-lowering, and blood pressure-lowering drugs), and surgical procedures (both open surgery and interventional therapy) (35). Despite these efforts, outcomes are often not as effective as desired, leading to significant declines in patients’ quality of life (6).

Exacerbated oxidative stress and reduced antioxidant capacity are important mechanisms underlying PAD. These factors contribute to endothelial dysfunction and ischemia–reperfusion injury by reducing nitric oxide (NO) production, while promoting atherosclerosis through the activation of NADPH oxidases and the release of proinflammatory cytokines and chemokines (7, 8). Consequently, controlling oxidative stress may represent a critical strategy for improving PAD outcomes. The Oxidative Balance Score (OBS) quantifies the body’s oxidative stress burden by evaluating dietary and lifestyle exposure to antioxidants relative to prooxidants (9). Reduced OBS has been linked to various conditions, including metabolic syndrome, diabetes mellitus, and cardiovascular disease, as well as increased all-cause, cancer, and cardiovascular mortality (10–13). Despite this, the exact relationship between OBS and PAD remains unclear, as there are limited studies that have extensively explored the impact of lifestyle and dietary factors on PAD. In this cross-sectional study, the association between OBS and PAD was examined, with the hypothesis that a higher OBS would be correlated with a decreased likelihood of developing PAD.

## 2 Materials and methods

### 2.1 Data sources and study population

The National Health and Nutrition Examination Survey (NHANES) is a comprehensive health survey conducted in the United States (36). It is administered by the Centers for Disease Control and Prevention (CDC) and uses a complex sampling design to collect a wide range of health data, including demographic information, physical exam results, lab tests, health questionnaires, and prescription drug details. The database is known for its representativeness and usefulness in research. All participants provided consent, and the National Center for Health Statistics (NCHS) Research Ethics Review Board approved the use of NHANES data, eliminating the need for additional ethical review for this study.

**Figure 1:**
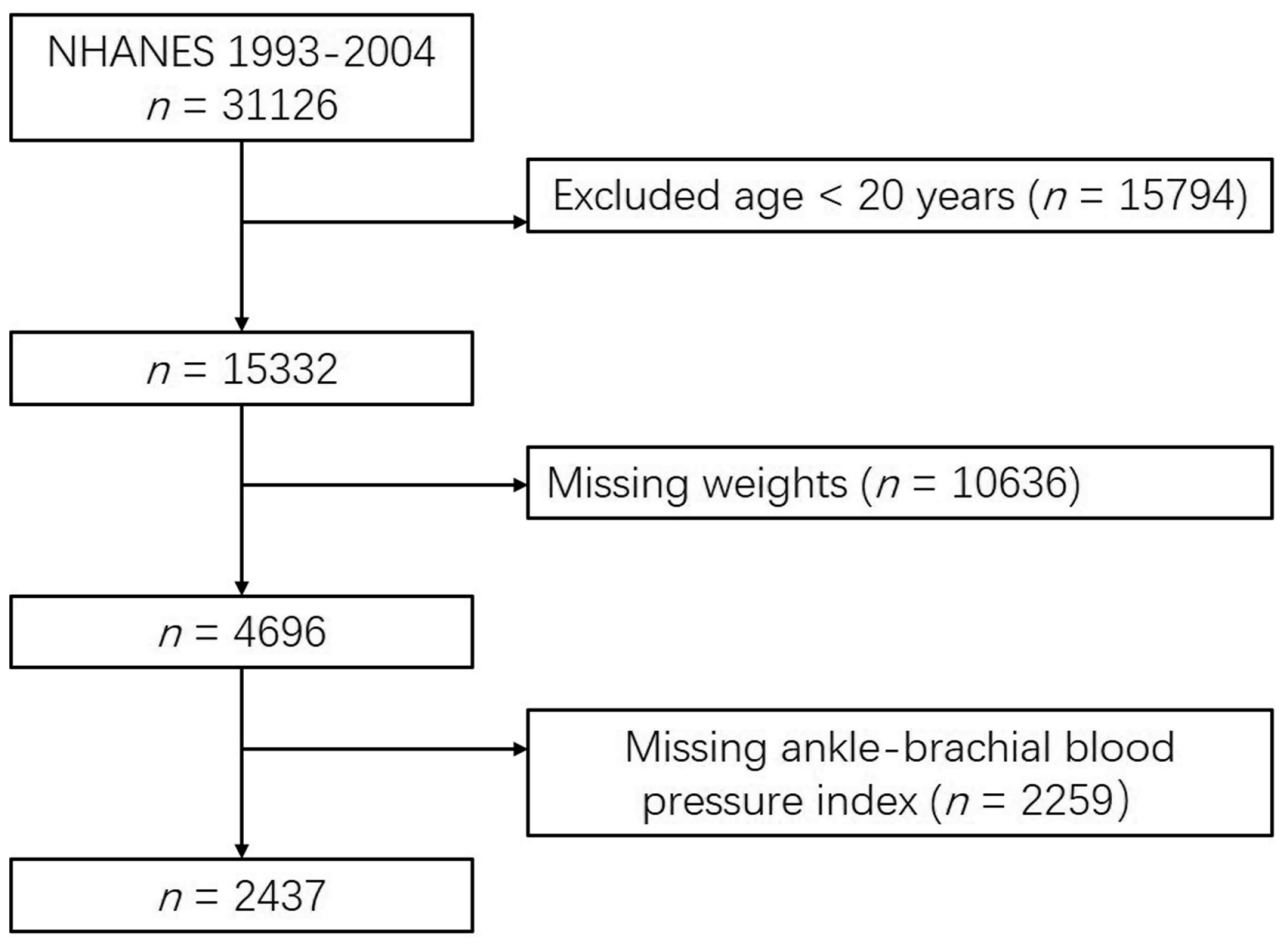
Flow diagram of the included survey participants.

Participants were selected from three consecutive cycles of NHANES data (1999–2004) for analysis. Eligibility criteria included individuals aged 20 years or older who were scheduled for an ankle-brachial index (ABI) test (*n* = 15,332). PAD was defined as an ABI ≤ 0.9. To minimize analytical bias, the following exclusions were applied: (1) participants with missing weight data (*n* = 10,636) and (2) participants with missing ABI data (*n* = 2,259). Ultimately, 2,437 eligible participants were included in the analysis (see Figure 1).

### 2.2 Outcome variable

An ABI ≤ 0.9 is the preferred non-invasive marker for diagnosing PAD (14). During the measurement, patients lie supine and remain still to minimize motion artifacts. Systolic blood pressures of the posterior tibial and brachial arteries are measured using a Doppler stethoscope, which accurately detects blood flow signals. The ABI is then calculated as the ratio of ankle systolic blood pressure to brachial systolic blood pressure. PAD is diagnosed when the ABI in at least one leg is ≤0.9 (5).

### 2.3 Exposure variable

The OBS calculation table was created using data from previous studies (37–39). The total OBS in this study included lifestyle OBS and dietary OBS. Dietary OBS consisted of 16 factors such as dietary fiber, carotenoids, and various vitamins and minerals. Lifestyle OBS included factors like drinking status, physical activity, and BMI. A 24-h dietary recall interview was conducted during the nutritional assessment phase after 2002, with the first interview done in person and the second over the phone. In the analysis of data from 2003–2004, the average of two recalls was used to represent intake. Out of the 20 components studied, total fat, iron, BMI, alcohol consumption, and smoking were categorized as prooxidants, while the rest were considered antioxidants. Each component was divided into three groups based on its distribution. Antioxidant scores ranged from 0 to 2, while prooxidant scores ranged from 2 to 0. The overall Oxidative Balance Score (OBS) was calculated by summing all component scores. Lifestyle OBS and dietary OBS were also calculated separately to examine their individual impacts on PAD. Higher OBS values indicated higher levels of antioxidant activity in participants (see Table 1).

**Table 1:** Total OBS assignment scheme (*n* = 2,437).

Total OBS components	Property	Male			Female		
		0	1	2	0	1	2
Dietary OBS components							
Dietary fiber (g/d)	A	<13.25	13.25–21.35	≥21.35	<11.55	11.55–17.50	≥17.50
Carotene (RE/d)	A	<580.00	580.00–1926.00	≥1926.00	<641.50	641.50–2247.00	≥2247.00
Riboflavin (mg/d)	A	<1.85	1.85–2.65	≥2.65	<1.47	1.47–2.08	≥2.08
Niacin (mg/d)	A	<23.39	23.39–33.66	≥33.66	<16.82	16.82–23.64	≥23.64
Vitamin B6 (mg/d)	A	<1.82	1.82–2.70	≥2.70	<1.32	1.32–1.93	≥1.93
Total folate (mcg/d)	A	<341.50	341.50–498.00	≥498.00	<272.00	272.00–388.00	≥388.00
Vitamin B12 (mcg/d)	A	<3.90	3.90–6.49	≥6.49	<2.71	2.71–4.69	≥4.69
Vitamin C (mg/d)	A	<38.90	38.90–98.55	≥98.55	<38.60	38.35–89.45	≥89.45
Vitamin E (ATE) (mg/d)	A	<6.19	6.19–9.60	≥9.60	<5.30	5.30–8.29	≥8.29
Calcium (mg/d)	A	<785.00	785.00–1209.50	≥1209.50	<658.50	658.50–980.00	≥980.00
Magnesium (mg/d)	A	<269.50	269.5–368.50	≥368.50	<200.00	200.00–301.50	≥301.50
Zinc (mg/d)	A	<10.21	10.21–14.71	≥14.71	<7.48	7.48–10.55	≥10.55
Copper (mg/d)	A	<1.07	1.07–1.52	≥1.52	<0.89	0.89–1.26	≥1.26
Selenium (mcg/d)	A	<100.45	100.45–146.90	≥146.90	<74.95	74.95–104.90	≥104.90
Total fat (g/d)	P	≥100.48	71.32–100.48	<71.32	≥76.29	53.05–76.29	<53.05
Iron (mg/d)	P	≥18.00	12.94–18.00	<12.94	≥14.08	9.91–14.08	<9.91
Dietary fiber (g/d)	A	<13.25	13.25–21.35	≥21.35	<11.55	11.55–17.50	≥17.50
Lifestyle OBS components							
Physical activity (MET-minute/week)	A	<1800.00	1800.00–5760.00	≥5760.00	<960.00	960.00–3240.00	≥3240.00
Alcohol (g/d)	P	>3	2–3	≤2	>2	1–2	≤1
Body mass index (kg/m^2^)	P	>29.16	25.54–29.16	<25.54	>28.64	23.74–28.64	<23.74
Cotinine (ng/mL)	P	>1.12	0.04–1.12	<0.04	>0.17	0.04–0.17	<0.04

Antioxidant was represented by A; pro-oxidant was represented by P; metabolic equivalent represented by MET; OBS, Oxidative balance score.

### 2.4 Covariates

Three categories of covariates were included in this study: sociodemographic information [Age, Sex, Race/ethnicity, Education, Poverty index ratio (PIR), Marital status], lifestyle behaviors (Smoking status, BMI), and chronic diseases (Heart disease, Hypertension, Stroke, Liver disease, Cancer). Participants aged 20 years or older were categorized by age as young adults (20–44 years), middle-aged adults (45–64 years), and older adults (≥65 years). Race/ethnicity was classified into Mexican American, Other Hispanic, Non-Hispanic White, Non-Hispanic Black, and Other Race/ethnicity – including Multi-Racial. Marital status was categorized as married/living with a partner, never married, or widowed/divorced. Educational attainment was classified into three groups: below high school, high school, and college or above. The PIR was categorized as <1, 1–1.99, 2–2.99, 3–3.99, and ≥ 4. Smoking status was divided into three categories: “current smoker,” including both daily and intermittent smokers; “former smoker,” for those who had smoked more than 100 cigarettes in their lifetime but were not current smokers; and “never smoker,” for those who had smoked fewer than 100 cigarettes in their lifetime. BMI was categorized into low to normal (<25 kg/m^2^), overweight (25–30 kg/m^2^), and obese (≥30 kg/m^2^). Chronic diseases, including hypertension, stroke, liver disease, heart disease, and cancer, were defined based on physician or health professional diagnosis. Heart disease was specified as congestive heart failure, coronary heart disease, angina pectoris, or other related cardiovascular conditions, according to questionnaire data.

### 2.5 Mediating variables

Our hypothesis is that Peripheral Arterial Disease (PAD) is influenced by various factors. Drawing from previous research, we have identified glucose-lipid metabolism and renal function indicators as potential mediators for the indirect impact of total obesity (OBS) on PAD (40, 41). Causal mediation analysis, rooted in the potential outcomes framework, allows us to break down the total exposure effect into causal direct and indirect effects, even when there are interactions between the exposure and the mediators. A key advantage of causal effect definitions is their nonparametric nature, making them applicable to any type of mediation model for estimating causal effects. In this study, the independent variable (x) is total OBS, the dependent variable (y) is PAD, and the mediating variables (M) include blood glucose, total cholesterol, triglycerides, creatinine, and urea nitrogen. Covariates adjusted for in the analysis include sex, age, race/ethnicity, education, income, marital status, smoking status, BMI, and the presence of heart disease, hypertension, stroke, liver disease, and cancer. The assessment indicators used are Total Effect (TE), Average Causal Mediation Effect (ACME), Average Direct Effect (ADE), and Proportional Mediation Effect (PM). The analysis involves three steps.

(1) Constructing a linear regression model for total OBS levels and glucose-lipid metabolism and renal function indicators.
(2) Constructing a logistic regression model for total OBS levels and PAD prevalence.
(3) Using the mediate function to calculate causal mediation effects.

### 2.6 Statistical analysis

Data for each subject in NHANES were collected by multiple departments and personnel, but various factors during the collection process resulted in unavoidable data loss. To address missing values in this study, random forest-based multiple imputation was utilized (15) (refer to Supplementary Table S1; Supplementary Figure S1). NHANES data were acquired through a complex sampling design, and this study included appropriate weighting in its descriptions and analyses.

In order to compare the characteristics of individuals with Peripheral Artery Disease (PAD) and those without PAD, *t*-tests or ANOVA were used to analyze differences. Continuous variables were reported as means ± standard deviation, while categorical variables were presented as frequencies and weighted percentages. Weighted logistic regression models were utilized to investigate the relationships between total OBS, lifestyle OBS, dietary OBS, and PAD. Model 1 was unadjusted (crude model), Model 2 was adjusted for Age and Sex, and Model 3 was adjusted for all covariates, including Sex, Age, Race/ethnicity, Education, PIR, Marital status, Smoking status, BMI, and the presence of Heart disease, Hypertension, Stroke, Liver disease, and Cancer.

Weighted restricted cubic spline (RCS) models were used to analyze the nonlinear associations of total OBS, lifestyle OBS, and dietary OBS with PAD and ABI. All covariates were adjusted for in the analysis. Stratified analyses were conducted based on Sex, Age, Race/ethnicity, Education, PIR, Marital status, Smoking status, BMI, and individual classifications of Heart disease, Hypertension, Stroke, Liver disease, and Cancer.

Two sensitivity analyses were conducted to assess the robustness of the findings. The first adjusted for hyperlipidemia and respiratory disease in addition to all covariates. The second evaluated the correlations between OBSs and PAD in a smaller unweighted sample to further validate the results.

All analyses were performed using R software (version 4.2.3^1^), with statistical significance set at *p* < 0.05.

## 3 Results

### 3.1 Participant characteristics

Table 2 displays the characteristics of individuals with PAD and those without PAD in this study. The data was gathered from three NHANES cycles (1999–2004), with a total of 2,437 participants included. This comprised 2,290 individuals without PAD and 147 individuals with PAD. In the PAD group, 58.3% were male and 41.7% were female. Additionally, 61.4% of individuals in the PAD group were aged 65 years or older.

**Table 2:** Baseline characteristics of NHANES participants (1999–2004) by PAD for OBS.

	non-PAD (*n* = 2,290)	PAD (*n* = 147)	*p*-value
Age, *n* (%)			
20–44	337 (18.6%)	1 (1.1%)	<0.001
45–64	1,192 (59.1%)	43 (37.5%)	
≥65	761 (22.2%)	103 (61.4%)	
Sex, *n* (%)			
Female	1,131 (51.7%)	74 (58.3%)	0.235
Male	1,159 (48.3%)	73 (41.7%)	
Race/ethnicity, *n* (%)			
Mexican American	525 (4.5%)	26 (3.0%)	0.234
Other Hispanic	101 (5.1%)	8 (5.8%)	
Non-Hispanic White	1,206 (77.8%)	80 (79.1%)	
Non-Hispanic Black	397 (8.8%)	31 (11.3%)	
Other Race – Including Multi-Racial	61 (3.8%)	2 (0.8%)	
Marital status, *n* (%)			
Married/living with partner	1,567 (72.7%)	82 (61.2%)	0.134
Never married	116 (4.8%)	7 (5.3%)	
Widowed/divorced	607 (22.5%)	58 (33.5%)	
Education, *n* (%)			
Below high school	777 (20.4%)	71 (40.3%)	<0.001
College or above	1,012 (54.5%)	45 (36.1%)	
High school	501 (25.1%)	31 (23.6%)	
PIR, *n* (%)			
<1	332 (10.0%)	36 (14.5%)	0.020
1–1.99	576 (18.0%)	39 (28.9%)	
2–3.99	626 (27.4%)	38 (27.5%)	
≥4	756 (44.5%)	34 (29.1%)	
Smoking, *n* (%)			
Current smoker	470 (21.3%)	40 (28.2%)	0.010
Former smoker	1,066 (45.7%)	45 (29.9%)	
Never smoker	754 (32.9%)	62 (41.9%)	
BMI, kg/m^2^, *n* (%)			
Low to normal (<25)	661 (31.3%)	49 (34.4%)	0.455
Obese (25–30)	743 (31.7%)	33 (26.5%)	
Overweight (≥30)	886 (37.0%)	65 (39.1%)	
Chronic disease, *n* (%)			
Hypertension	865 (34.0%)	86 (62.8%)	<0.001
Heart disease	242 (8.9%)	31 (22.3%)	<0.001
Cancer	249 (10.6%)	28 (18.0%)	0.031
Stroke	82 (3.0%)	16 (11.6%)	<0.001
Liver disease	99 (4.1%)	6 (3.1%)	0.574
Dietary OBS, *M* (SD)	13.99 (6.70)	11.72 (6.71)	0.003
Lifestyle OBS, *M* (SD)	2.36 (2.25)	1.70 (2.14)	<0.001
OBS, *M* (SD)	11.38 (10.34)	7.02 (9.05)	<0.001

*n* is unweighted values; % and *M* (SD) are weighted values. PAD, peripheral artery disease OBS, Oxidative balance score; PIR, poverty income ratio; BMI, body mass index.

Further analysis showed that individuals in the PAD group had significantly lower values of Dietary OBS, Lifestyle OBS, and total OBS compared to those in the non-PAD group (*p* < 0.05). This suggests a potential link between lower OBS levels and the development of PAD. Moreover, the incidence of PAD was significantly associated with Age, Education, PIR, Hypertension, Heart disease, Cancer, and Stroke. However, there were no statistically significant differences in Sex, Race/ethnicity, Marital status, BMI, or Liver disease (*p* > 0.05) (see Table 2).

### 3.2 Logistic regression analysis of the correlation between different OBS and PAD

Table 3 shows the logistic regression findings on the relationship between various levels of overall wellbeing score (OBS) and peripheral artery disease (PAD). In the initial model (Model 1), dietary OBS, lifestyle OBS, and total OBS were all found to have a negative correlation with PAD (*p* < 0.05). After adjusting for factors such as Age, Sex, Race/ethnicity, Marital status, Education, PIR, Smoking, BMI, Hypertension, Heart disease, Cancer, Stroke, and Liver disease, lifestyle OBS (OR = 0.88; 95% CI: 0.79, 1.00) and total OBS (OR = 0.97; 95% CI: 0.94, 0.99) continued to show a negative association with PAD. However, no significant link was observed between dietary OBS (OR = 0.97; 95% CI: 0.92, 1.02) and PAD (refer to Table 3 for details).

**Table 3:** Logistic regression model of different OBS and PAD.

Characteristic	Model 1			Model 2			Model 3		
	OR	95%CI	*p*-value	OR	95%CI	*p*-value	OR	95%CI	*p*-value
Dietary OBS	0.95	0.92, 0.98	0.006	0.96	0.92, 1.00	0.030	0.97	0.92, 1.02	0.180
Lifestyle OBS	0.87	0.80, 0.94	0.001	0.88	0.81, 0.95	0.002	0.88	0.79, 1.00	0.045
Total OBS	0.96	0.94, 0.97	<0.001	0.96	0.95, 0.98	<0.001	0.97	0.94, 0.99	0.009

Model 1: Unadjusted model (Crude model); Model 2: Adjusted for age, sex; Model 3: Adjusted for age, sex, Race/ethnicity, Marital status, Education, PIR, Smoking, BMI, Hypertension, Heart, Cancer, Stroke and Liver; PIR, poverty income ratio; BMI, body mass index; PAD, peripheral artery disease; OBS, Oxidative balance score; OR, Odds Ratio; CI, Confidence Interval.

### 3.3 Dose–response relationship of different OBS in association with PAD and ABI levels

**Figure 2:**
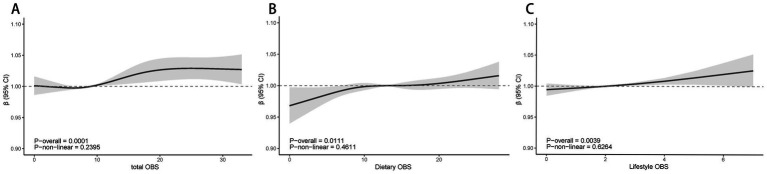
The dose–response association between total OBS(A), Dietary OBS **(A)**, Dietary OBS **(B)**, Lifestyle OBS **(C)** and PAD based on RCS analysis. Model 3: Adjusted for age, sex, Race/ethnicity, Marital status, Education, PIR, Smoking, BMI, Hypertension, Heart, Cancer, Stroke and Liver; PIR, poverty income ratio; BMI, body mass index; RCS, restricted cubic spline; OBS, Oxidative balance score; OR, Odds Ratio; CI, Confidence Interval.

Figure 2 demonstrates the nonlinear relationship between various levels of OBS and PAD. Through RCS analysis, which was conducted using weighted multivariate logistic regression and adjusted for covariates, it was found that there were no significant nonlinear associations between dietary OBS, lifestyle OBS, or total OBS with PAD. However, a linear correlation was observed between total OBS and PAD, showing a decrease in the likelihood of PAD as total OBS increased *p* for overall association = 0.0329, *p* for nonlinearity = 0.7355 (refer to Figure 2A).

**Figure 3:**
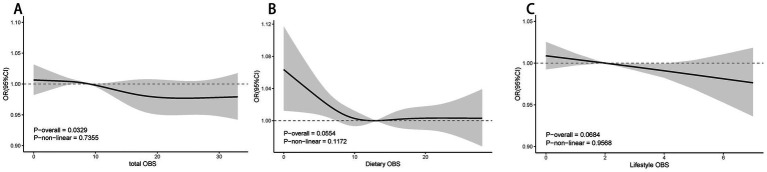
Multivariable-adjusted spline curves of relation between total OBS **(A)**, Dietary OBS **(B)**, Lifestyle OBS **(C)** and ABI. Model 3: Adjusted for age, sex, Race/ethnicity, Marital status, Education, PIR, Smoking, BMI, Hypertension, Heart, Cancer, Stroke and Liver; PIR, poverty income ratio; BMI, body mass index; RCS, restricted cubic spline; OBS, Oxidative balance score; ABI, ankle brachial index; CI, Confidence Interval.

Figure 3 illustrates the nonlinear correlation between different OBS levels and ABI. The results showed no significant nonlinear correlations for dietary OBS, lifestyle OBS, or total OBS with ABI. However, all three OBS types demonstrated positive linear correlations with ABI (*p* < 0.05) (see Figures 3A–C).

### 3.4 Mediation analysis

Previous research has demonstrated a strong association between renal injury (16) and glucose (17) with total OBS, both of which are also connected to peripheral arterial disease. This current study aimed to investigate whether total OBS indirectly impacts PAD through glycemic-lipid metabolism and renal function markers by conducting mediation analysis.

The study results showed that fasting glucose (FPG) and creatinine (CREA) played a significant role as mediators (*p* < 0.05) in the connection between total OBS and PAD. This suggests that total OBS could impact PAD risk indirectly by influencing blood sugar levels and kidney function. The average mediating effects of FPG and CREA were 5.9 and 0.8%, respectively, indicating a weaker but still statistically significant indirect influence of total OBS on PAD through these mechanisms (Table 4).

**Table 4:** Causal mediation analysis between total OBS and PAD.

	Direct effect (average)	*p*-value	Mediation effect (average)	*p*-value	Proportion-mediated (average)	*p*-value
FPG	−0.002 (−0.003,0.000)	<0.001	0.000 (0.000,0.000)	<0.001	0.059 (0.022,0.230)	<0.001
TC	−0.002 (−0.003,0.000)	0.010	0.000 (0.000,0.000)	0.720	−0.720 (−0.069,0.040)	0.710
TG	−0.002 (−0.003,0.000)	0.010	0.000 (0.000,0.000)	0.880	−0.001 (−0.059,0.030)	0.860
CREA	−0.002 (−0.003,0.000)	0.020	0.000 (0.000,0.000)	0.030	0.015 (0.002, 0.070)	0.040
BUN	−0.002 (−0.003,0.000)	0.020	0.000 (0.000,0.000)	0.440	0.008 (−0.019, 0.050)	0.440

Model 3: Adjusted for age, sex, Race/ethnicity, Marital status, Education, PIR, Smoking, BMI, Hypertension, Heart, Cancer, Stroke and Liver; PIR, poverty income ratio; BMI, body mass index; OBS, Oxidative balance score; PAD, peripheral artery disease; FPG, Fasting Plasma Glucose; TC, Total Cholesterol; TG, Triglyceride; CREA, creatinine; BUN, Blood Urea Nitrogen.

### 3.5 Sensitivity and subgroup analysis

Supplementary Tables S14–S18 present the results of the sensitivity analyses. After adjusting for hyperlipidemia and respiratory disease, total OBS remained negatively associated with PAD odds (OR = 0.97; 95% CI: 0.94, 0.99; *p =* 0.027). This finding suggests that the association between total OBS and PAD is highly stable. However, no statistically significant association was observed between lifestyle OBS and PAD incidence (*p* > 0.05), indicating that the lifestyle OBS results may lack stability after covariate adjustment and warrant further validation in future studies.

In a separate sensitivity analysis using unweighted data, total OBS (OR = 0.99; 95% CI: 0.98, 1.00; *p =* 0.004), dietary OBS (OR = 0.99; 95% CI: 0.98, 1.00; *p =* 0.032), and lifestyle OBS (OR = 0.94; 95% CI: 0.90, 0.99; *p =* 0.026) were all negatively associated with PAD. These findings indicate that total OBS, dietary OBS, and lifestyle OBS were significantly negatively associated with PAD incidence in the smaller sample data analysis.

**Figure 4:**
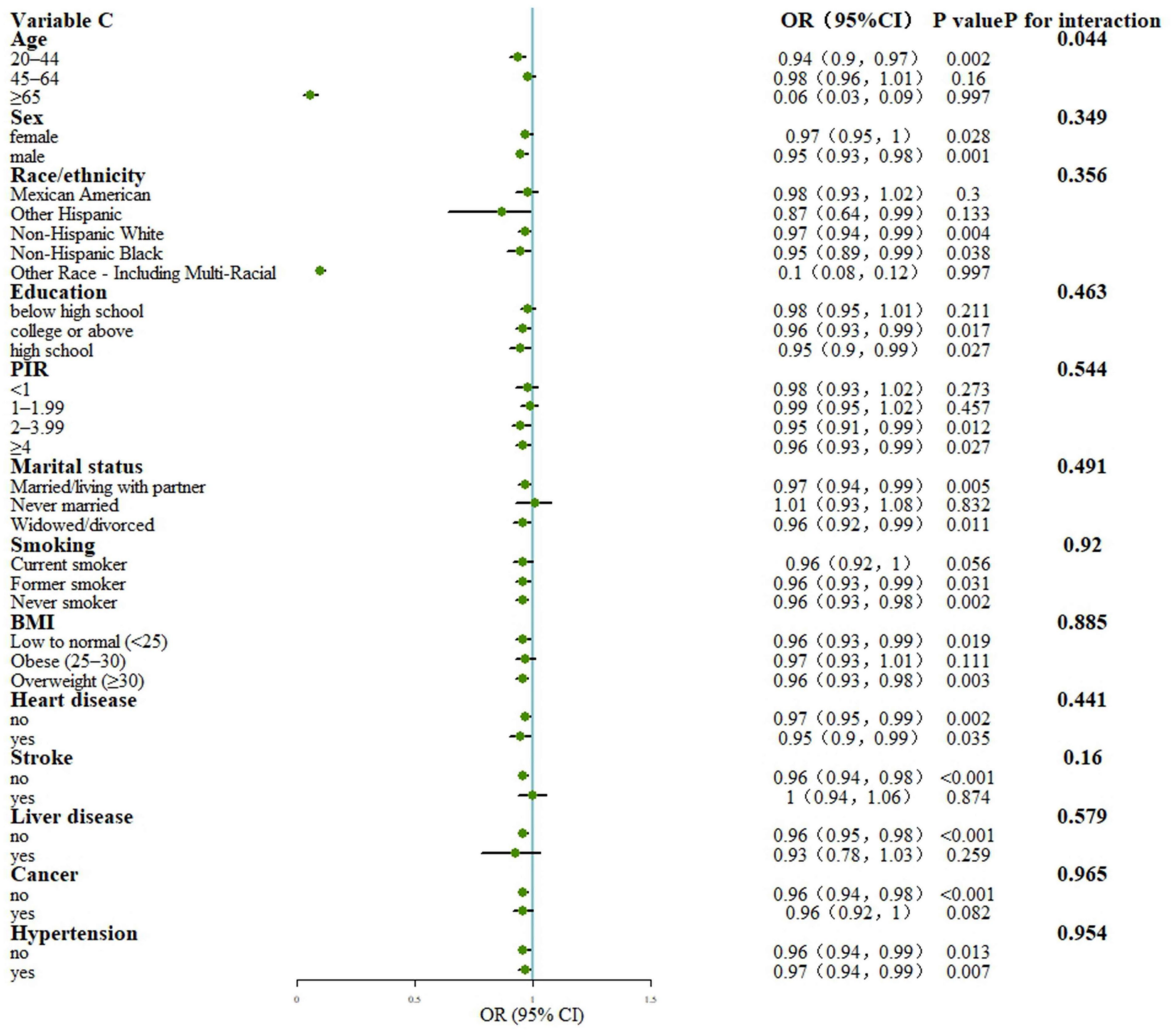
Stratified analysis for the association between OBS and PAD. Model: Adjusted for age, sex, Race/ethnicity, Marital status, Education, PIR, Smoking, BMI, Hypertension, Heart, Cancer, Stroke and Liver; PIR, poverty income ratio; BMI, body mass index; OBS, Oxidative balance score; OR, Odds Ratio; CI, Confidence Interval; PIR, poverty income ratio; BMI, body mass index.

The interaction test indicated that age played a significant role in influencing the relationship between total OBS and PAD (*p* < 0.05). Specifically, the strength of this association varied across different age groups. Upon conducting stratified analysis, it was found that there was a significant negative correlation between total OBS and PAD in the younger age group (20–44 years; *p* < 0.05), whereas this correlation was not significant in the older age groups (*p* > 0.05). These findings suggest that the link between total OBS and PAD is more pronounced in younger populations and may be influenced by different factors in older populations (see Figure 4).

## 4 Discussion

This study is the first to examine the associations of dietary OBS, lifestyle OBS, and total OBS with PAD. Our cross-sectional analysis of 2,437 participants from NHANES identified a significant negative association between total OBS and PAD. RCS analysis demonstrated a linear correlation between total OBS and PAD, indicating that higher total OBS levels (reflecting lower oxidative stress) were associated with a reduced likelihood of PAD. Additionally, RCS analysis revealed linear correlations between dietary OBS, lifestyle OBS, and total OBS with ABI. The results of the mediation analysis suggest that fasting plasma glucose (FPG) and creatinine (CREA) levels may play a role in mediating the relationship between total oxidative stress (OBS) and peripheral artery disease (PAD). This indicates that managing FPG and CREA levels could be important in reducing the risk of PAD. Sensitivity analyses supported these findings, showing that dietary OBS, lifestyle OBS, and total OBS were inversely associated with PAD in a smaller sample population. Stratified analyses also revealed that the relationship between total OBS and PAD was stronger in younger individuals (aged 20–44 years), suggesting that interventions targeting oxidative stress may be particularly beneficial for this age group.

The OBS is determined by adding together the scores for antioxidants and pro-oxidants. Research has demonstrated that physical activity increases blood circulation, improves the function of oxidative enzymes in muscles, and elevates the levels of antioxidants in the bloodstream of individuals with PAD (18). Among pro-oxidants, smoking exacerbates oxidative stress, producing harmful substances that alter blood lipid composition, thereby worsening atherosclerosis and contributing to PAD progression (19). Alcohol consumption also influences PAD risk. Individuals consuming more than 10 drinks per week have a significantly higher risk of developing PAD, while those consuming up to two drinks per week show the lowest risk (20). This may be due to the anti-inflammatory and oxidative stress-attenuating effects of moderate alcohol intake, which also improves microvascular function and peripheral atherosclerosis. These pro-oxidant factors significantly increase PAD risk (21), highlighting the potential benefits of improving lifestyle behaviors to reduce PAD likelihood (22). Various components of dietary OBS, such as *β*-carotene, vitamin B6, dietary fiber, vitamin B12, vitamin C, vitamin D, folate, copper, calcium, selenium, and magnesium, have antioxidant effects and are associated with PAD risk (23, 24). This aligns with our findings, where RCS analyses demonstrated a significant negative linear association between total OBS and PAD. Dietary OBS showed significant associations in sensitivity analyses conducted on smaller sample populations. Lifestyle OBS remained significantly associated with PAD after adjusting for all variables, suggesting a more stable and essential role for lifestyle in PAD management. The relationship between BMI and oxidative stress is well established (25), but the independent association between BMI and PAD remains controversial (26). One study found no significant association between BMI and PAD, with the lowest PAD prevalence observed in individuals with a BMI of 25–29.9 kg/m^2^, while a BMI ≥30 kg/m^2^ was associated with an increased risk of PAD only in women (27). Conversely, another study linked a BMI ≥30 kg/m^2^ combined with adipose tissue dysfunction and body fat distribution abnormalities (e.g., high body fat percentage, abdominal obesity, and excessive white adipose tissue) to a higher PAD risk (26). Factors such as genetic inheritance, menopausal characteristics, and the protective properties of different adipose tissues may explain these discrepancies. Our hypothesis suggests that the development of PAD is closely linked to oxidative stress, which is characterized by an increase in free radicals and a decrease in both endogenous and exogenous antioxidant capacity. This oxidative stress state may be affected by various factors such as lifestyle choices, genetic predisposition, and other variables. It is possible to enhance antioxidant capacity through dietary and lifestyle changes, including exogenous supplementation. These factors may interact with each other, as seen in the influence of exercise, total fat intake, and dietary fiber intake on BMI (28).

Additionally, a single antioxidant (e.g., vitamin E) alone may not sufficiently alleviate PAD symptoms (29). However, a combination of multiple antioxidants (e.g., sodium, potassium, selenium, magnesium) derived from antioxidant-rich fruits and vegetables can synergistically enhance antioxidant effects, alleviating PAD symptoms (30). While examining the impact of individual pro-oxidants or antioxidants on PAD may not fully capture the oxidative stress-PAD relationship, this does not diminish the importance of dietary OBS in this context. Our study demonstrated that total OBS is significantly and negatively associated with PAD in a linear manner. Dietary components, which constitute a substantial proportion of total OBS, play a central role in this association. Specific dietary OBS components, such as *β*-carotene, vitamin C, vitamin E, and magnesium, have potent antioxidant effects and may reduce PAD by mitigating oxidative stress-induced arterial endothelial damage. Thus, this study underscores the importance of dietary OBS as not only a measure of the body’s oxidative stress burden but also as a potential target for reducing PAD through dietary improvements and increased antioxidant intake.

Oxidative stress is characterized by an imbalance between excessive reactive oxygen species (ROS) and the limited functionality of antioxidant defense systems or enzymatic dysfunction (31). It plays a key role in the pathogenesis of PAD and contributes to metabolic disorders, hypertension, and vascular endothelial damage through mechanisms such as mitochondrial complex dysfunction, NADPH oxidase activity, and e-NOS dysregulation (32). Oxidative stress in the body can be addressed to potentially prevent and slow down the development of PAD. The study evaluated oxidative balance by looking at a total OBS score based on 20 different dietary and lifestyle factors. A lower OBS score indicates higher levels of oxidative stress. Weighted logistic regression analysis revealed a strong negative link between total OBS score and the likelihood of developing PAD, and additional analyses confirmed the reliability of this finding. Stratified analyses revealed that age significantly moderated this association, with a stronger negative correlation observed in younger age groups (20–44 years). This suggests that age may act as a moderator in the relationship between total OBS and PAD, likely because oxidative stress levels and PAD risk are both age-dependent factors. Mediation analyses indicated that fasting plasma glucose (FPG) and creatinine (CREA) partially mediate the relationship between total OBS and PAD. Elevated blood glucose levels are known to increase oxidative stress and impair vascular health (33). Similarly, renal insufficiency is strongly linked to increased oxidative stress and vascular injury (34). These findings highlight the importance of glycemic and renal function management in reducing oxidative stress and managing PAD. This study also explores factors influencing oxidative stress levels, building on previous literature (22). For instance, increased physical activity and the intake of antioxidant-rich vitamins and micronutrients (e.g., vitamin C, selenium, magnesium) significantly enhance antioxidant capacity and boost OBS values. On the other hand, decreasing smoking, alcohol intake, BMI, and body fat can decrease oxidative stress and improve oxidative balance score (OBS). While these lifestyle changes may impact the likelihood of peripheral artery disease (PAD), more research is needed to understand the specific mechanisms and effects. Our results highlight the strong negative relationship between total OBS and PAD, indicating that dietary and lifestyle changes could indirectly reduce the risk of PAD by enhancing oxidative balance. However, further studies and trials are necessary to better understand how these factors influence PAD risk reduction through improved oxidative balance.

There are several strengths to our study. Firstly, it is the first nationwide research to examine the connections between dietary oxidative balance score (OBS), lifestyle OBS, and total OBS with peripheral artery disease (PAD). Secondly, the association between OBS and PAD was confirmed using various statistical methods, increasing the clinical significance and trustworthiness of the results. Thirdly, by investigating the oxidative/antioxidant dietary and lifestyle factors linked to PAD, the study offers further evidence for evaluating PAD risk. These findings can be valuable in predicting PAD and providing strategic insights for its prevention and treatment.

While this study has several strengths, it is important to acknowledge its limitations. Firstly, the cross-sectional design does not allow for establishing a causal relationship between oxidative homeostasis and PAD, only associations. Secondly, PAD was defined solely based on ABI ≤ 0.9 without further categorization into specific subtypes, potentially underestimating the heterogeneity of the disease. Despite adjusting for various covariates, the influence of unknown confounders cannot be completely ruled out. Additionally, differences in dietary habits and lifestyles across countries may limit the generalizability of the findings. Future long-term follow-up studies should refine PAD classification, conduct subgroup analyses, and explore potential causal relationships in diverse populations. Validating these results in different regions is crucial to ensure their broader applicability.

## 5 Conclusion

This study found a significant negative association between total OBS, a measure of oxidative stress burden, and the prevalence of PAD. Higher levels of total OBS were linked to a reduced likelihood of PAD. The mediation analysis indicated that controlling blood glucose levels (e.g., FPG) and renal function (e.g., CREA) plays a crucial role in the relationship between total OBS and PAD. These results underscore the potential benefits of antioxidant-rich diets and healthy lifestyle habits in managing PAD risk, especially in younger populations (e.g., 20–44 years old).

## Data Availability

The datasets presented in this study can be found in online repositories. The names of the repository/repositories and accession number(s) can be found in the article/Supplementary material.

## Footnotes

^1^ www.r-project.org
