# Supplementary material for: Association between oxidative balance scores and peripheral artery disease in US adults: a cross-sectional study
Source: Front Nutr. 2025 Jan 17;11:1497784. doi: 10.3389/fnut.2024.1497784 (PMC11782040; doi:10.3389/fnut.2024.1497784)
Supplement: Supplementary file 1 [file Supplementary_file_1.docx]

Supplementary Table 1. Missing data patterns

| **Pattern** | ***N*** | **%** |
| --- | --- | --- |
| DMDEDUC2 | 6 | 0.25 |
| INDFMPIR | 258 | 10.59 |
| DMDMARTL | 111 | 4.55 |
| SMO | 3 | 0.12 |
| BMXBMI | 37 | 1.52 |
| MCQ160B | 8 | 0.33 |
| MCQ160C | 21 | 0.86 |
| MCQ160D | 12 | 0.49 |
| MCQ160E | 7 | 0.29 |
| MCQ160F | 4 | 0.16 |
| MCQ160L | 7 | 0.29 |
| MCQ220 | 1 | 0.04 |
| BPQ020 | 13 | 0.53 |
| DIQ010 | 40 | 1.64 |
| LBXGLUSI | 98 | 4.02 |
| LBXGH | 73 | 3.00 |
| LBXSCR | 126 | 5.17 |
| LBXSCH | 125 | 5.13 |
| LBXSBU | 126 | 5.17 |
| LBXSTR | 125 | 5.13 |

Supplementary Table 2. Unweighted baseline table

|  | non-PAD  (*n* = 2290) | PAD  (*n* = 147) | PAD  (*n* = 2437) | *p*-value |
| --- | --- | --- | --- | --- |
| Age, *M (SD)* | | | | |
| 20–44 | 337 (14.7%) | 1 (0.7%) | 338 (13.9%) | <0.001 |
| 45–64 | 1192 (52.1%) | 43 (29.3%) | 1235 (50.7%) |  |
| ≥65 | 761 (33.2%) | 103 (70.1%) | 864 (35.5%) |  |
| Sex, *n* (%) | | | | |
| female | 1131 (49.4%) | 74 (50.3%) | 1205 (49.4%) | 0.975 |
| male | 1159 (50.6%) | 73 (49.7%) | 1232 (50.6%) |  |
| Race/ethnicity, *n* (%) | | | | |
| Mexican American | 525 (22.9%) | 26 (17.7%) | 551 (22.6%) | 0.848 |
| Other Hispanic | 101 (4.4%) | 8 (5.4%) | 109 (4.5%) |  |
| Non-Hispanic White | 1206 (52.7%) | 80 (54.4%) | 1286 (52.8%) |  |
| Non-Hispanic Black | 397 (17.3%) | 31 (21.1%) | 428 (17.6%) |  |
| Other Race - Including Multi-Racial | 61 (2.7%) | 2 (1.4%) | 63 (2.6%) |  |
| Marital status, *n* (%) | | | | |
| Married/living with partner | 1567 (68.4%) | 82 (55.8%) | 1649 (67.7%) | 0.0191 |
| Never married | 116 (5.1%) | 7 (4.8%) | 123 (5.0%) |  |
| Widowed/divorced | 607 (26.5%) | 58 (39.5%) | 665 (27.3%) |  |
| Education, *n* (%) | | | | |
| below high school | 777 (33.9%) | 71 (48.3%) | 848 (34.8%) | 0.00696 |
| college or above | 1012 (44.2%) | 45 (30.6%) | 1057 (43.4%) |  |
| high school | 501 (21.9%) | 31 (21.1%) | 532 (21.8%) |  |
| PIR, *n* (%) | | | | |
| <1 | 332 (14.5%) | 36 (24.5%) | 368 (15.1%) | 0.0356 |
| 1–1.99 | 576 (25.2%) | 39 (26.5%) | 615 (25.2%) |  |
| 2–3.99 | 626 (27.3%) | 38 (25.9%) | 664 (27.2%) |  |
| ≥4 | 756 (33.0%) | 34 (23.1%) | 790 (32.4%) |  |
| Smoking, *n* (%) | | | | |
| Current smoker | 470 (20.5%) | 40 (27.2%) | 510 (20.9%) | 0.00675 |
| Former smoker | 1066 (46.6%) | 45 (30.6%) | 1111 (45.6%) |  |
| Never smoker | 754 (32.9%) | 62 (42.2%) | 816 (33.5%) |  |
| BMI, kg/m^2^, *n* (%) | | | | |
| Low to normal (<25) | 661 (28.9%) | 49 (33.3%) | 710 (29.1%) | 0.174 |
| Obese (25–30) | 743 (32.4%) | 33 (22.4%) | 776 (31.8%) |  |
| Overweight (≥30) | 886 (38.7%) | 65 (44.2%) | 951 (39.0%) |  |
| Chronic disease, *n* (%) | | | | |
| Hypertension | 865 (37.8%) | 86 (58.5%) | 951 (39.0%) | <0.001 |
| Heart disease | 242 (10.6%) | 31 (21.1%) | 273 (11.2%) | <0.001 |
| Cancer | 249 (10.9%) | 28 (19.0%) | 277 (11.4%) | 0.0102 |
| Stroke | 82 (3.6%) | 16 (10.9%) | 98 (4.0%) | <0.001 |
| Liver disease | 99 (4.3%) | 6 (4.1%) | 105 (4.3%) | 0.99 |
| Dietary OBS, *M (SD)* | 13.3 (6.74) | 11.6 (6.85) | 13.2 (6.76) | 0.0139 |
| Lifestyle OBS, *M (SD)* | 2.07 (2.19) | 1.56 (2.14) | 2.04 (2.19) | 0.00942 |
| OBS, *M (SD)* | 9.92 (10.1) | 6.49 (8.93) | 9.71 (10.1) | <0.001 |

*Note: n is unweighted values; % and M (SD) are weighted values; PAD: peripheral artery disease; PIR:* *poverty income ratio; BMI: body mass index;* *OBS: oxidative balance score.*

Supplementary Table 3. Logistic regression analysis of dietary OBS and PAD

| Characteristic | OR | 95% CI | *p*-value |
| --- | --- | --- | --- |
| Sex | | | |
| female | — | — |  |
| male | 0.88 | 0.56, 1.38 | 0.6 |
| Age | | | |
| ≥65 | — | — |  |
| 20–44 | 0.02 | 0.00, 0.21 | 0.002 |
| 45–64 | 0.23 | 0.14, 0.40 | <0.001 |
| Dietary OBS | 0.96 | 0.92, 1.00 | 0.030 |

*Note:* *model 2: Adjusted for age, sex; OBS: oxidative balance score; PAD: peripheral artery disease; OR: Odds Ratio; CI: Confidence Interval.*

Supplementary Table 4. Logistic regression analysis of lifestyle OBS with PAD

| Characteristic | OR | 95% CI | *p*-value |
| --- | --- | --- | --- |
| Sex | | | |
| female | — | — |  |
| male | 0.85 | 0.53, 1.36 | 0.5 |
| Age | | | |
| ≥65 | — | — |  |
| 20–44 | 0.02 | 0.00, 0.19 | 0.001 |
| 45–64 | 0.24 | 0.14, 0.40 | <0.001 |
| Lifestyle OBS | 0.88 | 0.81, 0.95 | 0.002 |

*Note: model 2: Adjusted for age, sex; OBS: oxidative balance score; PAD: peripheral artery disease; OR: Odds Ratio; CI: Confidence Interval.*

Supplementary Table 5. Logistic regression analysis of total OBS with PAD

| Characteristic | OR | 95% CI | *p*-value |
| --- | --- | --- | --- |
| Sex | | | |
| female | — | — |  |
| male | 0.91 | 0.58, 1.44 | 0.7 |
| Age | | | |
| ≥65 | — | — |  |
| 20–44 | 0.02 | 0.00, 0.21 | 0.002 |
| 45–64 | 0.24 | 0.14, 0.41 | <0.001 |
| OBS | 0.96 | 0.95, 0.98 | <0.001 |

*Note: model 2: Adjusted for age, sex; OBS: oxidative balance score; PAD: peripheral artery disease; OR: Odds Ratio; CI: Confidence Interval.*

Supplementary Table 6. Logistic regression analysis of dietary OBS with PAD

| Characteristic | OR | 95% CI | *p*-value |
| --- | --- | --- | --- |
| Sex | | | |
| female | — | — |  |
| male | 0.78 | 0.41, 1.47 | 0.4 |
| Age | | | |
| ≥65 | — | — |  |
| 20–44 | 0.03 | 0.00, 0.56 | 0.027 |
| 45–64 | 0.29 | 0.13, 0.60 | 0.008 |
| Race | | | |
| Mexican American | — | — |  |
| Other Hispanic | 1.10 | 0.18, 6.58 | 0.9 |
| Non-Hispanic White | 1.62 | 0.53, 4.93 | 0.3 |
| Non-Hispanic Black | 1.66 | 0.63, 4.37 | 0.2 |
| Other Race - Including Multi-Racial | 0.48 | 0.06, 4.09 | 0.4 |
| Education | | | |
| below high school | — | — |  |
| college or above | 0.52 | 0.28, 0.97 | 0.043 |
| high school | 0.50 | 0.21, 1.22 | 0.10 |
| PIR | | | |
| <1 | — | — |  |
| 1–1.99 | 1.07 | 0.43, 2.69 | 0.9 |
| 2–3.99 | 1.01 | 0.28, 3.63 | >0.9 |
| ≥4 | 1.05 | 0.38, 2.87 | >0.9 |
| Marriage | | | |
| Married/living with partner | — | — |  |
| Never married | 1.72 | 0.28, 10.6 | 0.5 |
| Widowed/divorced | 1.03 | 0.48, 2.21 | >0.9 |
| Smoke | | | |
| Current smoker | — | — |  |
| Former smoker | 0.42 | 0.19, 0.93 | 0.038 |
| Never smoker | 0.76 | 0.34, 1.67 | 0.4 |
| BMI, kg/m^2^ | | | |
| Low to normal (<25) | — | — |  |
| Obese (≥30) | 0.66 | 0.29, 1.52 | 0.3 |
| Overweight (25–30) | 0.92 | 0.54, 1.58 | 0.7 |
| Heart | | | |
| no | — | — |  |
| yes | 1.34 | 0.76, 2.37 | 0.2 |
| Stroke | | | |
| no | — | — |  |
| yes | 1.75 | 0.64, 4.79 | 0.2 |
| Liver | | | |
| no | — | — |  |
| yes | 0.78 | 0.16, 3.74 | 0.7 |
| Cancer | | | |
| no | — | — |  |
| yes | 1.00 | 0.49, 2.07 | >0.9 |
| Hypertension | | | |
| no | — | — |  |
| yes | 2.35 | 1.16, 4.76 | 0.026 |
| Dietary OBS | 0.97 | 0.92, 1.02 | 0.2 |

*Note: Model 3: Adjusted for age, sex, Race/ethnicity, Marital status, Education, PIR, Smoking, BMI, Hypertension, Heart, Cancer, Stroke and Liver; OBS: oxidative balance score; PAD: peripheral artery disease; PIR: poverty income ratio; BMI: body mass index; OR: Odds Ratio; CI: Confidence Interval.*

Supplementary Table 7. Logistic regression analysis of lifestyle OBS with PAD

| Characteristic | OR | 95% CI*^1^* | *p*-value |
| --- | --- | --- | --- |
| Sex | | | |
| female | — | — |  |
| male | 0.73 | 0.37, 1.45 | 0.3 |
| Age | | | |
| ≥65 | — | — |  |
| 20–44 | 0.03 | 0.00, 0.54 | 0.026 |
| 45–64 | 0.28 | 0.14, 0.57 | 0.006 |
| Race | | | |
| Mexican American | — | — |  |
| Other Hispanic | 1.15 | 0.19, 7.09 | 0.8 |
| Non-Hispanic White | 1.65 | 0.55, 4.94 | 0.3 |
| Non-Hispanic Black | 1.68 | 0.64, 4.44 | 0.2 |
| Other Race - Including Multi-Racial | 0.47 | 0.05, 4.11 | 0.4 |
| Education | | | |
| below high school | — | — |  |
| college or above | 0.51 | 0.27, 0.94 | 0.037 |
| high school | 0.50 | 0.21, 1.18 | 0.094 |
| PIR | | | |
| <1 | — | — |  |
| 1–1.99 | 1.10 | 0.45, 2.71 | 0.8 |
| 2–3.99 | 1.06 | 0.29, 3.85 | >0.9 |
| ≥4 | 1.15 | 0.41, 3.22 | 0.7 |
| Marriage | | | |
| Married/living with partner | — | — |  |
| Never married | 1.67 | 0.30, 9.28 | 0.5 |
| Widowed/divorced | 0.97 | 0.43, 2.16 | >0.9 |
| Smoke | | | |
| Current smoker | — | — |  |
| Former smoker | 0.39 | 0.18, 0.87 | 0.029 |
| Never smoker | 0.73 | 0.33, 1.62 | 0.4 |
| BMI, kg/m^2^ | | | |
| Low to normal (<25) | — | — |  |
| Obese (≥30) | 0.57 | 0.24, 1.35 | 0.2 |
| Overweight (25–30) | 0.83 | 0.47, 1.48 | 0.4 |
| Heart | | | |
| no | — | — |  |
| yes | 1.42 | 0.79, 2.56 | 0.2 |
| Stroke | | | |
| no | — | — |  |
| yes | 1.55 | 0.52, 4.62 | 0.4 |
| Liver | | | |
| no | — | — |  |
| yes | 0.69 | 0.16, 3.04 | 0.5 |
| Cancer | | | |
| no | — | — |  |
| yes | 1.03 | 0.51, 2.08 | >0.9 |
| Hypertension | | | |
| no | — | — |  |
| yes | 2.34 | 1.16, 4.71 | 0.026 |
| Lifestyle OBS | 0.88 | 0.79, 1.00 | 0.045 |

*Note: Model 3: Adjusted for age, sex, Race/ethnicity, Marital status, Education, PIR, Smoking, BMI, Hypertension, Heart, Cancer, Stroke and Liver; PIR: poverty income ratio; BMI: body mass index; OBS: oxidative balance score; PAD: peripheral artery disease; OR: Odds Ratio; CI: Confidence Interval.*

Supplementary Table 8. Logistic Regression Analysis of total OBS and the PAD

| Characteristic | OR | 95% CI | *p*-value |
| --- | --- | --- | --- |
| Sex | | | |
| female | — | — |  |
| male | 0.77 | 0.41, 1.48 | 0.4 |
| Age | | | |
| ≥65 | — | — |  |
| 20–44 | 0.03 | 0.00, 0.57 | 0.027 |
| 45–64 | 0.28 | 0.14, 0.58 | 0.006 |
| Race | | | |
| Mexican American | — | — |  |
| Other Hispanic | 1.10 | 0.18, 6.79 | 0.9 |
| Non-Hispanic White | 1.63 | 0.54, 4.90 | 0.3 |
| Non-Hispanic Black | 1.61 | 0.61, 4.28 | 0.3 |
| Other Race - Including Multi-Racial | 0.44 | 0.05, 3.82 | 0.4 |
| Education | | | |
| below high school | — | — |  |
| college or above | 0.53 | 0.28, 0.97 | 0.044 |
| high school | 0.51 | 0.22, 1.20 | 0.10 |
| PIR | | | |
| <1 | — | — |  |
| 1–1.99 | 1.09 | 0.44, 2.68 | 0.8 |
| 2–3.99 | 1.06 | 0.29, 3.84 | >0.9 |
| ≥4 | 1.18 | 0.42, 3.31 | 0.7 |
| Marriage | | | |
| Married/living with partner | — | — |  |
| Never married | 1.72 | 0.29, 10.2 | 0.5 |
| Widowed/divorced | 0.99 | 0.45, 2.15 | >0.9 |
| Smoke | | | |
| Current smoker | — | — |  |
| Former smoker | 0.38 | 0.17, 0.83 | 0.025 |
| Never smoker | 0.72 | 0.33, 1.60 | 0.3 |
| BMI, kg/m^2^ | | | |
| Low to normal (<25) | — | — |  |
| Obese (≥30) | 0.61 | 0.27, 1.38 | 0.2 |
| Overweight (25–30) | 0.88 | 0.50, 1.54 | 0.6 |
| Heart | | | |
| no | — | — |  |
| yes | 1.36 | 0.75, 2.45 | 0.2 |
| Stroke | | | |
| no | — | — |  |
| yes | 1.57 | 0.53, 4.69 | 0.3 |
| Liver | | | |
| no | — | — |  |
| yes | 0.68 | 0.15, 3.11 | 0.5 |
| Cancer | | | |
| no | — | — |  |
| yes | 1.00 | 0.49, 2.02 | >0.9 |
| Hypertension | | | |
| no | — | — |  |
| yes | 2.37 | 1.17, 4.78 | 0.025 |
| OBS | 0.97 | 0.94, 0.99 | 0.009 |

*Note: Model 3: Adjusted for age, sex, Race/ethnicity, Marital status, Education, PIR, Smoking, BMI, Hypertension, Heart, Cancer, Stroke and Liver; PIR: poverty income ratio; BMI: body mass index; OBS: Oxidative balance score; PAD: peripheral artery disease; OR: Odds Ratio; CI: Confidence Interval.*

Supplementary Table 9. Mediation analysis of FPG in the relationship between total OBS and PAD

| Type | Estimate | 95% CI Lower | 95% CI Upper | *p*-value |
| --- | --- | --- | --- | --- |
| ACME (control) | 0.000 | 0.000 | 0.000 | <0.001 |
| ACME (treated) | 0.000 | 0.000 | 0.000 | <0.001 |
| ADE (control) | -0.002 | -0.003 | 0.000 | <0.001 |
| ADE (treated) | -0.002 | -0.003 | 0.000 | <0.001 |
| Total Effect | -0.002 | -0.003 | 0.000 | <0.001 |
| Proportion mediated (control) | 0.060 | 0.022 | 0.230 | <0.001 |
| Proportion mediated (treated) | 0.059 | 0.022 | 0.230 | <0.001 |
| ACME (Average) | 0.000 | 0.000 | 0.000 | <0.001 |
| ADE (Average) | -0.002 | -0.003 | 0.000 | <0.001 |
| Proportion mediated | 0.059 | 0.022 | 0.230 | <0.001 |

*Note: Model 3: Adjusted for age, sex, Race/ethnicity, Marital status, Education, PIR, Smoking, BMI, Hypertension, Heart, Cancer, Stroke and Liver; PIR: poverty income ratio; BMI: body mass index; FPG: fasting plasma glucose; OBS: oxidative balance score; PAD: peripheral artery disease; ACME: average causal mediation effect; ADE: average direct effect; Proportion mediated= ACME/Total effect; CI: confidence interval.*

Supplementary Table 10. Mediation analysis of TC in the relationship between total OBS and PAD

| Type | Estimate | 95% CI Lower | 95% CI Upper | *p*-value |
| --- | --- | --- | --- | --- |
| ACME (control) | 0.000 | 0.000 | 0.000 | 0.720 |
| ACME (treated) | 0.000 | 0.000 | 0.000 | 0.720 |
| ADE (control) | -0.002 | -0.003 | 0.000 | 0.010 |
| ADE (treated) | -0.002 | -0.003 | 0.000 | 0.010 |
| Total Effect | -0.002 | -0.003 | 0.000 | 0.010 |
| Proportion mediated (control) | -0.007 | -0.069 | 0.040 | 0.710 |
| Proportion mediated (treated) | -0.007 | -0.068 | 0.040 | 0.710 |
| ACME (Average) | 0.000 | 0.000 | 0.000 | 0.720 |
| ADE (Average) | -0.002 | -0.003 | 0.000 | 0.010 |
| Proportion mediated | -0.007 | -0.069 | 0.040 | 0.710 |

*Note: Model 3: Adjusted for age, sex, Race/ethnicity, Marital status, Education, PIR, Smoking, BMI, Hypertension, Heart, Cancer, Stroke and Liver; PIR: poverty income ratio; BMI: body mass index; TC: total cholesterol; OBS: oxidative balance score; PAD: peripheral artery disease; ACME: average causal mediation effect; ADE: average direct effect; Proportion mediated= ACME/Total effect; CI: confidence interval.*

Supplementary Table 11. Mediation analysis of TG in the relationship between total OBS and PAD

| Type | Estimate | 95% CI Lower | 95% CI Upper | *p*-value |
| --- | --- | --- | --- | --- |
| ACME (control) | 0.000 | 0.000 | 0.000 | 0.880 |
| ACME (treated) | 0.000 | 0.000 | 0.000 | 0.880 |
| ADE (control) | -0.002 | -0.003 | 0.000 | 0.020 |
| ADE (treated) | -0.002 | -0.003 | 0.000 | 0.020 |
| Total Effect | -0.002 | -0.003 | 0.000 | 0.020 |
| Proportion mediated (control) | -0.001 | -0.060 | 0.030 | 0.860 |
| Proportion mediated (treated) | -0.001 | -0.059 | 0.030 | 0.860 |
| ACME (Average) | 0.000 | 0.000 | 0.000 | 0.880 |
| ADE (Average) | -0.002 | -0.003 | 0.000 | 0.020 |
| Proportion mediated | -0.001 | -0.059 | 0.030 | 0.860 |

*Note: Model 3: Adjusted for age, sex, Race/ethnicity, Marital status, Education, PIR, Smoking, BMI, Hypertension, Heart, Cancer, Stroke and Liver; PIR: poverty income ratio; BMI: body mass index; TG: triglyceride; OBS: oxidative balance score; PAD: peripheral artery disease; ACME: average causal mediation effect; ADE: average direct effect; Proportion mediated= ACME/Total effect; CI: confidence interval.*

Supplementary Table 12. Causal mediation analysis of CREA in the relationship between total OBS and PAD

| Type | Estimate | 95% CI Lower | 95% CI Upper | *p*-value |
| --- | --- | --- | --- | --- |
| ACME (control) | 0.000 | 0.000 | 0.000 | 0.030 |
| ACME (treated) | 0.000 | 0.000 | 0.000 | 0.030 |
| ADE (control) | -0.002 | -0.003 | 0.000 | 0.020 |
| ADE (treated) | -0.002 | -0.003 | 0.000 | 0.020 |
| Total Effect | -0.002 | -0.003 | 0.000 | 0.010 |
| Proportion mediated (control) | 0.016 | 0.002 | 0.080 | 0.040 |
| Proportion mediated (treated) | 0.015 | 0.002 | 0.070 | 0.040 |
| ACME (Average) | 0.000 | 0.000 | 0.000 | 0.030 |
| ADE (Average) | -0.002 | -0.003 | 0.000 | 0.020 |
| Proportion mediated | 0.015 | 0.002 | 0.070 | 0.040 |

*Note: Model 3: Adjusted for age, sex, Race/ethnicity, Marital status, Education, PIR, Smoking, BMI, Hypertension, Heart, Cancer, Stroke and Liver; PIR: poverty income ratio; BMI: body mass index; CREA: creatinine; OBS: oxidative balance score; PAD: peripheral artery disease; ACME: average causal mediation effect; ADE: average direct effect; Proportion mediated= ACME/Total effect; CI: confidence interval.*

Supplementary Table 13. Causal mediation analysis of BUN in the relationship between total OBS and PAD

| Type | Estimate | 95% CI Lower | 95% CI Upper | *p*-value |
| --- | --- | --- | --- | --- |
| ACME (control) | 0.000 | 0.000 | 0.000 | 0.440 |
| ACME (treated) | 0.000 | 0.000 | 0.000 | 0.440 |
| ADE (control) | -0.002 | -0.003 | 0.000 | 0.020 |
| ADE (treated) | -0.002 | -0.003 | 0.000 | 0.020 |
| Total Effect | -0.002 | -0.003 | 0.000 | <0.001 |
| Proportion mediated (control) | 0.008 | -0.019 | 0.050 | 0.440 |
| Proportion mediated (treated) | 0.008 | -0.019 | 0.050 | 0.440 |
| ACME (Average) | 0.000 | 0.000 | 0.000 | 0.440 |
| ADE (Average) | -0.002 | -0.003 | 0.000 | 0.020 |
| Proportion mediated | 0.008 | -0.019 | 0.050 | 0.440 |

*Note: Model 3: Adjusted for age, sex, Race/ethnicity, Marital status, Education, PIR, Smoking, BMI, Hypertension, Heart, Cancer, Stroke and Liver; PIR: poverty income ratio; BMI: body mass index; BUN: blood urea nitrogen; OBS: oxidative balance score; PAD: peripheral artery disease; ACME: average causal mediation effect; ADE: average direct effect; Proportion mediated= ACME/Total effect; CI: confidence interval.*

Supplementary Table 14. Total OBS sensitivity analysis (hyperlipidemia, respiratory diseases)

| Characteristic | OR | 95% CI | *p*-value |
| --- | --- | --- | --- |
| Sex | | | |
| female | — | — |  |
| male | 0.77 | 0.35, 1.73 | 0.4 |
| Age | | | |
| ≥65 | — | — |  |
| 20–44 | 0.03 | 0.00, 1.11 | 0.054 |
| 45–64 | 0.28 | 0.12, 0.68 | 0.020 |
| Race | | | |
| Mexican American | — | — |  |
| Other Hispanic | 1.06 | 0.11, 9.81 | >0.9 |
| Non-Hispanic White | 1.58 | 0.39, 6.38 | 0.4 |
| Non-Hispanic Black | 1.61 | 0.48, 5.39 | 0.3 |
| Other Race - Including Multi-Racial | 0.41 | 0.03, 6.37 | 0.4 |
| Education | | | |
| below high school | — | — |  |
| college or above | 0.53 | 0.24, 1.18 | 0.086 |
| high school | 0.51 | 0.17, 1.48 | 0.14 |
| PIR | | | |
| <1 | — | — |  |
| 1–1.99 | 1.09 | 0.35, 3.35 | 0.8 |
| 2–3.99 | 1.05 | 0.21, 5.32 | >0.9 |
| ≥4 | 1.21 | 0.32, 4.58 | 0.7 |
| Marriage | | | |
| Married/living with partner | — | — |  |
| Never married | 1.75 | 0.20, 15.6 | 0.5 |
| Widowed/divorced | 0.99 | 0.38, 2.58 | >0.9 |
| Smoke | | | |
| Current smoker | — | — |  |
| Former smoker | 0.39 | 0.14, 1.13 | 0.067 |
| Never smoker | 0.75 | 0.28, 1.99 | 0.4 |
| BMI, kg/m^2^ | | | |
| Low to normal (<25) | — | — |  |
| Obese (≥30) | 0.58 | 0.19, 1.78 | 0.2 |
| Overweight (25–30) | 0.86 | 0.42, 1.74 | 0.5 |
| Heart | | | |
| no | — | — |  |
| yes | 1.30 | 0.64, 2.65 | 0.3 |
| Stroke | | | |
| no | — | — |  |
| yes | 1.54 | 0.38, 6.32 | 0.4 |
| Liver | | | |
| no | — | — |  |
| yes | 0.69 | 0.11, 4.32 | 0.6 |
| Cancer | | | |
| no | — | — |  |
| yes | 0.99 | 0.40, 2.45 | >0.9 |
| Hypertension | | | |
| no | — | — |  |
| yes | 2.38 | 1.00, 5.69 | 0.050 |
| Hyperlipidemia | | | |
| no | — | — |  |
| yes | 1.14 | 0.47, 2.78 | 0.7 |
| Respiratory disease | | | |
| no | — | — |  |
| yes | 1.34 | 0.35, 5.09 | 0.5 |
| OBS | 0.97 | 0.94, 0.99 | 0.027 |

*Note: Model 3(+hyperlipidemia and respiratory disease): Adjusted for age, sex, Race/ethnicity, Marital status, Education, PIR, Smoking, BMI, Hypertension, Heart, Cancer, Stroke, Liver, hyperlipidemia and respiratory disease; PIR: poverty income ratio; BMI: body mass index; OBS: Oxidative balance score; OR: Odds Ratio; CI: Confidence Interval.*

Supplementary Table 15. Lifestyle OBS sensitivity analysis (hyperlipidemia, respiratory disease)

| Characteristic | Log (OR) | 95% CI | *p*-value |
| --- | --- | --- | --- |
| Sex | | | |
| female | — | — |  |
| male | 0.73 | 0.31, 1.71 | 0.3 |
| Age | | | |
| ≥65 | — | — |  |
| 20–44 | 0.03 | 0.00, 1.07 | 0.052 |
| 45–64 | 0.28 | 0.11, 0.67 | 0.019 |
| Race | | | |
| Mexican American | — | — |  |
| Other Hispanic | 1.11 | 0.12, 10.2 | 0.9 |
| Non-Hispanic White | 1.61 | 0.40, 6.42 | 0.4 |
| Non-Hispanic Black | 1.68 | 0.51, 5.59 | 0.3 |
| Other Race - Including Multi-Racial | 0.44 | 0.03, 6.90 | 0.4 |
| Education | | | |
| below high school | — | — |  |
| college or above | 0.51 | 0.23, 1.14 | 0.076 |
| high school | 0.50 | 0.17, 1.47 | 0.13 |
| PIR | | | |
| <1 | — | — |  |
| 1–1.99 | 1.10 | 0.35, 3.41 | 0.8 |
| 2–3.99 | 1.05 | 0.21, 5.32 | >0.9 |
| ≥4 | 1.18 | 0.31, 4.46 | 0.7 |
| Marriage | | | |
| Married/living with partner | — | — |  |
| Never married | 1.69 | 0.20, 13.9 | 0.5 |
| Widowed/divorced | 0.97 | 0.36, 2.62 | >0.9 |
| Smoke | | | |
| Current smoker | — | — |  |
| Former smoker | 0.41 | 0.14, 1.17 | 0.073 |
| Never smoker | 0.75 | 0.28, 2.01 | 0.4 |
| BMI, kg/m^2^ | | | |
| Low to normal (<25) | — | — |  |
| Obese (≥30) | 0.54 | 0.17, 1.75 | 0.2 |
| Overweight (25–30) | 0.81 | 0.39, 1.67 | 0.4 |
| Heart | | | |
| no | — | — |  |
| yes | 1.36 | 0.67, 2.75 | 0.3 |
| Stroke | | | |
| no | — | — |  |
| yes | 1.52 | 0.37, 6.24 | 0.4 |
| Liver | | | |
| no | — | — |  |
| yes | 0.70 | 0.12, 4.18 | 0.6 |
| Cancer | | | |
| no | — | — |  |
| yes | 1.02 | 0.42, 2.52 | >0.9 |
| Hypertension | | | |
| no | — | — |  |
| yes | 2.35 | 0.99, 5.60 | 0.052 |
| Hyperlipidemia | | | |
| no | — | — |  |
| yes | 1.15 | 0.47, 2.81 | 0.7 |
| Respiratory disease | | | |
| no | — | — |  |
| yes | 1.33 | 0.35, 5.08 | 0.5 |
| Lifestyle_OBS | 0.88 | 0.76, 1.03 | 0.082 |

*Note: Model 3(+hyperlipidemia and respiratory disease): Adjusted for age, sex, Race/ethnicity, Marital status, Education, PIR, Smoking, BMI, Hypertension, Heart, Cancer, Stroke, Liver, hyperlipidemia and respiratory disease; PIR: poverty income ratio; BMI: body mass index; OBS: Oxidative balance score; OR: Odds Ratio; CI: Confidence Interval.*

Supplementary Table 16. Total OBS sensitivity analysis (unweighted)

| **Characteristic** | **OR** | **95% CI** | ***p*-value** |
| --- | --- | --- | --- |
| **Sex** |  |  |  |
| female | — | — |  |
| male | 0.83 | 0.67, 1.04 | 0.10 |
| **Age** |  |  |  |
| ≥65 | — | — |  |
| 20–44 | 0.09 | 0.04, 0.17 | <0.001 |
| 45–64 | 0.31 | 0.24, 0.40 | <0.001 |
| **Race** |  |  |  |
| Mexican American | — | — |  |
| Other Hispanic | 0.87 | 0.44, 1.61 | 0.7 |
| Non-Hispanic White | 1.38 | 1.02, 1.88 | 0.040 |
| Non-Hispanic Black | 1.65 | 1.18, 2.32 | 0.004 |
| Other Race - Including Multi-Racial | 0.91 | 0.41, 1.83 | 0.8 |
| **Education** |  |  |  |
| below high school | — | — |  |
| college or above | 0.88 | 0.67, 1.14 | 0.3 |
| high school | 0.99 | 0.76, 1.29 | >0.9 |
| **PIR** |  |  |  |
| <1 | — | — |  |
| 1–1.99 | 0.88 | 0.66, 1.18 | 0.4 |
| 2–3.99 | 0.77 | 0.56, 1.05 | 0.10 |
| ≥4 | 0.61 | 0.43, 0.88 | 0.008 |
| **Marriage** |  |  |  |
| Married/living with partner | — | — |  |
| Never married | 0.84 | 0.47, 1.41 | 0.5 |
| Widowed/divorced | 1.28 | 1.02, 1.60 | 0.030 |
| **Smoke** |  |  |  |
| Current smoker | — | — |  |
| Former smoker | 0.39 | 0.29, 0.52 | <0.001 |
| Never smoker | 0.70 | 0.53, 0.92 | 0.009 |
| **BMI**, kg/m^2^ |  |  |  |
| Low to normal (<25) | — | — |  |
| Obese (≥30) | 0.91 | 0.70, 1.18 | 0.5 |
| Overweight (25–30) | 0.99 | 0.78, 1.26 | >0.9 |
| **Heart** |  |  |  |
| no | — | — |  |
| yes | 1.56 | 1.23, 1.97 | <0.001 |
| **Stroke** |  |  |  |
| no | — | — |  |
| yes | 1.95 | 1.39, 2.69 | <0.001 |
| **Liver** |  |  |  |
| no | — | — |  |
| yes | 0.94 | 0.55, 1.52 | 0.8 |
| **Cancer** |  |  |  |
| no | — | — |  |
| yes | 1.10 | 0.84, 1.43 | 0.5 |
| **Hypertension** |  |  |  |
| no | — | — |  |
| yes | 1.60 | 1.30, 1.98 | <0.001 |
| **OBS** | 0.99 | 0.98, 1.00 | 0.004 |

*Note: Model 3: Adjusted for age, sex, Race/ethnicity, Marital status, Education, PIR, Smoking, BMI, Hypertension, Heart, Cancer, Stroke and Liver; PIR: poverty income ratio; BMI: body mass index; OBS: Oxidative balance score; OR: Odds Ratio; CI: Confidence Interval.*

Supplementary Table 17. Dietary OBS sensitivity analysis (unweighted)

| **Characteristic** | **OR** | **95% CI** | ***p*-value** |
| --- | --- | --- | --- |
| **Sex** |  |  |  |
| female | — | — |  |
| male | 0.82 | 0.66, 1.02 | 0.079 |
| **Age** |  |  |  |
| ≥65 | — | — |  |
| 20–44 | 0.09 | 0.04, 0.17 | <0.001 |
| 45–64 | 0.31 | 0.24, 0.39 | <0.001 |
| **Race** |  |  |  |
| Mexican American | — | — |  |
| Other Hispanic | 0.85 | 0.42, 1.57 | 0.6 |
| Non-Hispanic White | 1.39 | 1.02, 1.89 | 0.037 |
| Non-Hispanic Black | 1.66 | 1.19, 2.34 | 0.003 |
| Other Race - Including Multi-Racial | 0.94 | 0.42, 1.89 | 0.9 |
| **Education** |  |  |  |
| below high school | — | — |  |
| college or above | 0.87 | 0.66, 1.13 | 0.3 |
| high school | 0.98 | 0.75, 1.28 | 0.9 |
| **PIR** |  |  |  |
| <1 | — | — |  |
| 1–1.99 | 0.89 | 0.67, 1.18 | 0.4 |
| 2–3.99 | 0.75 | 0.55, 1.03 | 0.076 |
| ≥4 | 0.58 | 0.41, 0.83 | 0.003 |
| **Marriage** |  |  |  |
| Married/living with partner | — | — |  |
| Never married | 0.83 | 0.46, 1.39 | 0.5 |
| Widowed/divorced | 1.29 | 1.03, 1.61 | 0.026 |
| **Smoke** |  |  |  |
| Current smoker | — | — |  |
| Former smoker | 0.41 | 0.31, 0.54 | <0.001 |
| Never smoker | 0.71 | 0.54, 0.94 | 0.014 |
| **BMI**, kg/m^2^ |  |  |  |
| Low to normal (<25) | — | — |  |
| Obese (≥30) | 0.93 | 0.71, 1.21 | 0.6 |
| Overweight (25–30) | 0.99 | 0.78, 1.27 | >0.9 |
| **Heart** |  |  |  |
| no | — | — |  |
| yes | 1.57 | 1.24, 1.98 | <0.001 |
| **Stroke** |  |  |  |
| no | — | — |  |
| yes | 2.00 | 1.43, 2.76 | <0.001 |
| **Liver** |  |  |  |
| no | — | — |  |
| yes | 0.97 | 0.57, 1.57 | >0.9 |
| **Cancer** |  |  |  |
| no | — | — |  |
| yes | 1.12 | 0.85, 1.45 | 0.4 |
| **Hypertension** |  |  |  |
| no | — | — |  |
| yes | 1.61 | 1.31, 2.00 | <0.001 |
| **Dietary_OBS** | 0.99 | 0.98, 1.00 | 0.032 |

*Note: Model 3: Adjusted for age, sex, Race/ethnicity, Marital status, Education, PIR, Smoking, BMI, Hypertension, Heart, Cancer, Stroke and Liver; PIR: poverty income ratio; BMI: body mass index; OBS: Oxidative balance score; OR: Odds Ratio; CI: Confidence Interval.*

Supplementary Table 18. Lifestyle OBS sensitivity analysis (unweighted)

| **Characteristic** | **OR** | **95% CI** | ***p*-value** |
| --- | --- | --- | --- |
| **Sex** |  |  |  |
| female | — | — |  |
| male | 0.81 | 0.65, 1.01 | 0.062 |
| **Age** |  |  |  |
| ≥65 | — | — |  |
| 20–44 | 0.09 | 0.04, 0.17 | <0.001 |
| 45–64 | 0.31 | 0.24, 0.39 | <0.001 |
| **Race** |  |  |  |
| Mexican American | — | — |  |
| Other Hispanic | 0.87 | 0.43, 1.61 | 0.7 |
| Non-Hispanic White | 1.39 | 1.03, 1.89 | 0.037 |
| Non-Hispanic Black | 1.67 | 1.20, 2.35 | 0.003 |
| Other Race - Including Multi-Racial | 0.93 | 0.41, 1.86 | 0.8 |
| **Education** |  |  |  |
| below high school | — | — |  |
| college or above | 0.86 | 0.66, 1.12 | 0.3 |
| high school | 0.97 | 0.74, 1.27 | 0.8 |
| **PIR** |  |  |  |
| <1 | — | — |  |
| 1–1.99 | 0.88 | 0.66, 1.17 | 0.4 |
| 2–3.99 | 0.76 | 0.56, 1.04 | 0.085 |
| ≥4 | 0.60 | 0.42, 0.86 | 0.005 |
| **Marriage** |  |  |  |
| Married/living with partner | — | — |  |
| Never married | 0.84 | 0.47, 1.41 | 0.5 |
| Widowed/divorced | 1.28 | 1.03, 1.60 | 0.029 |
| **Smoke** |  |  |  |
| Current smoker | — | — |  |
| Former smoker | 0.40 | 0.30, 0.53 | <0.001 |
| Never smoker | 0.71 | 0.54, 0.94 | 0.015 |
| **BMI**, kg/m^2^ |  |  |  |
| Low to normal (<25) | — | — |  |
| Obese (≥30) | 0.88 | 0.67, 1.14 | 0.3 |
| Overweight (25–30) | 0.97 | 0.76, 1.23 | 0.8 |
| **Heart** |  |  |  |
| no | — | — |  |
| yes | 1.57 | 1.23, 1.98 | <0.001 |
| **Stroke** |  |  |  |
| no | — | — |  |
| yes | 1.95 | 1.39, 2.69 | <0.001 |
| **Liver** |  |  |  |
| no | — | — |  |
| yes | 0.94 | 0.55, 1.52 | 0.8 |
| **Cancer** |  |  |  |
| no | — | — |  |
| yes | 1.11 | 0.84, 1.44 | 0.5 |
| **Hypertension** |  |  |  |
| no | — | — |  |
| yes | 1.60 | 1.29, 1.98 | <0.001 |
| **Lifestyle_OBS** | 0.94 | 0.90, 0.99 | 0.026 |

*Note: Model 3: Adjusted for age, sex, Race/ethnicity, Marital status, Education, PIR, Smoking, BMI, Hypertension, Heart, Cancer, Stroke and Liver; PIR: poverty income ratio; BMI: body mass index; OBS: Oxidative balance score; OR: Odds Ratio; CI: Confidence Interval.*


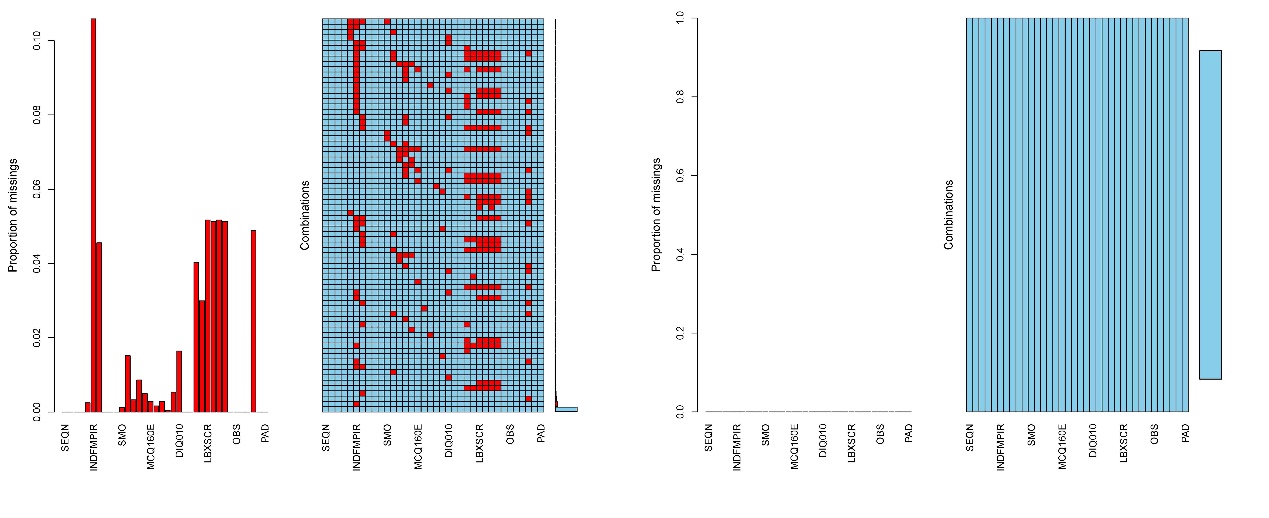


Supplementary Figure 1. Data situation diagram before and after multiple imputation
